# Transarterial chemoembolization with rivoceranib and camrelizumab for BCLC stage C hepatocellular carcinoma

**DOI:** 10.3389/fonc.2025.1710686

**Published:** 2025-12-10

**Authors:** Rui Zhu, Zi-Xuan Zhang, Xin-Zhi Lu, Kui Mao

**Affiliations:** 1 Department of Interventional Radiology, The First People’s Hospital of Linhai, Linhai, China; 2 Department of Radiology, Xuzhou Central Hospital, Xuzhou, China; 3 Department of General Surgery, The First People’s Hospital of Linhai, Linhai, China

**Keywords:** transarterial chemoembolization, camrelizumab, rivoceranib, hepatocellular carcinoma, intervention

## Abstract

**Background:**

Transarterial chemoembolization (TACE) and systemic therapy are frequently applied for advanced hepatocellular carcinoma (HCC), but their combined therapeutic potential remains uncertain. Here, the clinical efficacy and safety of TACE together with rivoceranib plus camrelizumab in Barcelona Clinic Liver Cancer (BCLC) stage C HCC were investigated.

**Methods:**

Between January 2021 and December 2024, 167 consecutive cases of BCLC stage C HCC were retrospectively analyzed. Eighty-three received rivoceranib-camrelizumab with TACE, and 84 received rivoceranib-camrelizumab alone. Comparisons of baseline characteristics, tumor response, long-term outcomes, and adverse events were conducted.

**Results:**

Baseline variables were balanced between groups. Combination therapy achieved significantly higher partial (39.8% vs. 20.2%, P = 0.006) and objective response rates (50.6% vs. 29.7%, P = 0.006) than systemic therapy alone. Median progression-free survival (PFS: 11.0 vs. 9.0 months, P = 0.008) and overall survival (OS: 19.0 vs. 15.0 months, P = 0.001) were both longer in the combination group. Cox regression identified combined treatment as an independent predictor of extended PFS and OS. Post-embolization symptoms occurred only in the TACE group but no grade 3-4 events were observed, and rates of systemic treatment-related adverse events were comparable between groups.

**Conclusions:**

Adding TACE to rivoceranib-camrelizumab has the potential to improve tumor response and survival in BCLC stage C HCC without compromising safety.

## Introduction

Despite its global prevalence, the curative resection of hepatocellular carcinoma (HCC) lesions is feasible in only about 20% of patients (1, 2). In 2023, the Asia-Pacific HCC Trials Group showed that 7% to44% of HCC patients presented with Barcelona Clinic Liver Cancer (BCLC) stage C disease at diagnosis in the Asia-Pacific regions (3). Among the Asia-Pacific regions, China had the highest incident rate (44%) of BCLC stage C HCC (3). According to BCLC guidelines, systemic therapy is the recommended first-line approach for these patients (4–6).

Contemporary systemic regimens for HCC increasingly combine tyrosine kinase inhibitors (TKIs) with immune checkpoint inhibitors (4–6). Until recently, sorafenib and lenvatinib were the standard first-line TKIs in this context (6). However, in 2023, the CARES-310 trial demonstrated that rivoceranib plus camrelizumab significantly improved both progression-free survival (PFS: 5.6 vs. 3.7 months, P < 0.001) and overall survival (OS: 22.1 vs. 15.2 months, P < 0.001) in comparison with sorafenib in unresectable HCC (6), establishing this combination as a novel first-line standard (6).

Despite advances in systemic therapy, local interventions remain central to HCC management (7). Transarterial chemoembolization (TACE) is the most frequently employed locoregional therapy (7), and recent studies have suggested additive benefits when TACE is combined with lenvatinib or lenvatinib-camrelizumab (4, 5). However, whether TACE can similarly enhance outcomes with rivoceranib-camrelizumab remains untested.

Here, the efficacy and safety of the combination of TACE and rivoceranib-camrelizumab in BCLC stage C HCC were investigated.

## Methods

### Study design

This single-center study retrospective received approval from the The First People’s Hospital of Linhai City Ethics Committee (Linyilunshen-2024-002) and performed as per the Declaration of Helsinki. Given its retrospective nature, written informed consent requirements for this study were waived.

From January 2021 to December 2024, we included 167 consecutive patients with BCLC stage C HCC who received rivoceranib-camrelizumab with (n = 83) or without TACE (n = 84). The patients chose combined treatment or rivoceranib-camrelizumab alone treatment based on their family economic condition. Baseline demographics, treatment response, survival outcomes, and adverse events were recorded.

Inclusion criteria were: (a) confirmed BCLC stage C HCC; (b) Child-Pugh class A or B liver function; (c) Eastern Cooperative Oncology Group (ECOG) performance status 0-1; and (d) at least one measurable lesion per modified Response Evaluation Criteria in Solid Tumors (mRECIST) criteria. Exclusion criteria included: (a) age <18 or >80 years; (b) prior anticancer therapy for HCC; (c) concomitant malignancy; or (d) complete main-portal-vein tumor thrombus because this is the contraindication for TACE.

American Association for the Study of Liver Diseases guidelines were used for HCC diagnosis (8). Collected data included age, sex, hepatitis status, liver function tests, ECOG score, imaging findings, treatment response, PFS, OS, and adverse events.

### Treatments and follow-up

In the combination group, conventional TACE was performed first (details in Supplementary Materials), followed approximately 10 days later by rivoceranib-camrelizumab therapy. Patients in the monotherapy group received rivoceranib-camrelizumab alone.

All patients were monitored at 1, 3, and 6 months, then every 3 months until death, loss to follow-up, or June 30, 2025. Subsequent interventions including ablation, radiotherapy, or alternative systemic therapies were permitted after tumor progression. Follow-up assessments involved complete blood counts, coagulation profiling, tests for liver and kidney function, serum electrolytes, and computed tomography (CT) or magnetic resonance imaging (MRI) scans. Tumor response was evaluated as per the mRECIST criteria. The first evaluation of tumor response was performed at 1 month, then every 3 months after treatment with the contrast-medium enhanced CT and/or MRI. PFS represented the interval between the start of treatment and documented progression, death, or final follow-up, while OS was measured from initiation of treatment to death or final follow-up. Adverse events were graded according to the National Cancer Institute Common Terminology Criteria (version 5.0).

### Statistical analyses

Means ± standard deviation or medians (interquartile range) are used when reporting continuous variables, which were compared as appropriate with t-tests or Mann-Whitney U tests, while categorical variables were assessed with χ² or Fisher’s exact tests. PFS and OS were evaluated with Kaplan-Meier curves and log-rank tests. Risk factors for OS were explored via a Cox regression approach, in which factors with P < 0.1 in the univariate analyses were retained for multivariate analysis. SPSS 16.0 was used for all statistical testing.

## Results

### Participant characteristics

**Figure 1:**
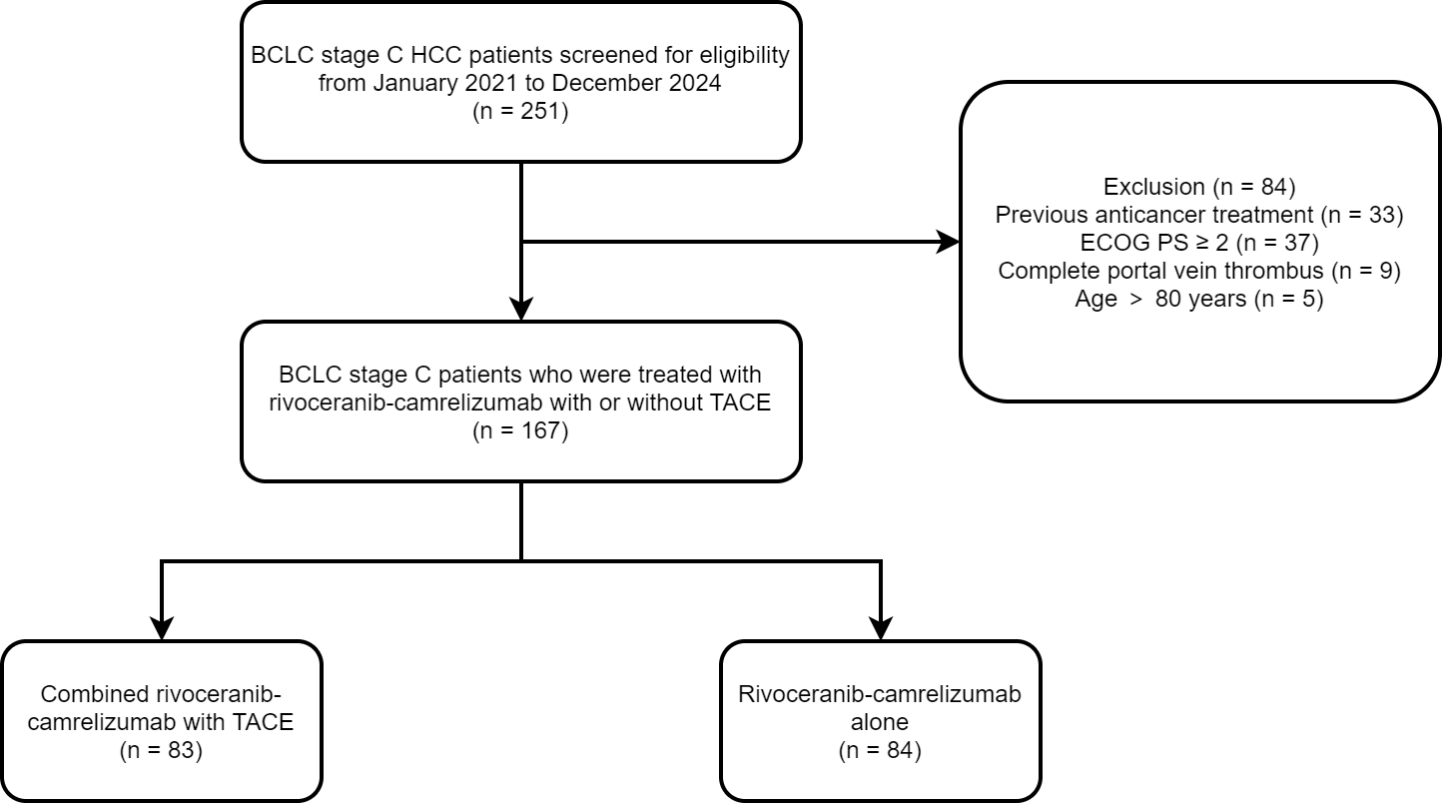
The flowchart of this study.

Baseline clinical data for all 167 patients are presented in **Table 1**, and the patient selection process is depicted in **Figure 1**. Demographic and clinical characteristics were well balanced between the cohorts, indicating no significant baseline differences.

**Table 1:** Baseline data of the included patients.

Variables	Combined group	Rivoceranib-camrelizumab alone group	P value
Patients number	83	84	Not applicable
Age (y)	59.9 ± 8.8	61.4 ± 8.0	0.234
Gender			0.454
Male	69	66	
Female	14	18	
Hepatitis			0.293
B	57	49	
C	1	3	
None	25	32	
Hepatocirrhosis	47	47	0.930
ECOG PS			0.389
0	48	43	
1	35	41	
Child-Pugh score	5.8 ± 0.8	5.9 ± 1.4	0.407
ALBI grade			0.551
1	26	33	
2	52	46	
3	5	5	
AFP value (mg/L)	41.2 (4.5-2000.0)	36.8 (4.3-625.5)	0.226
Extra-hepatic metastasis	18	22	0.495
Vascular invasion	70	74	0.481
Number of tumors	–		0.293
1	34	25	
2	35	40	
≥ 3	14	19	
Largest tumor size (mm)	6.7 ± 3.2	5.8 ± 3.8	0.095

AFP, alpha-fetoprotein; ALBI, Albumin-Bilirubin grade; ECOG PS, Eastern Cooperative Oncology Group performance status; HCC, hepatocellular carcinoma.

### Treatment response

Treatment response outcomes are detailed in **Table 2**. Compared with rivoceranib-camrelizumab alone, the combination of TACE with rivoceranib-camrelizumab resulted in a markedly higher partial response rate (39.8% vs. 20.2%, P = 0.006) and a superior overall objective response rate (50.6% vs. 29.7%, P = 0.006). Rates of complete response (10.8% vs. 9.5%, P = 0.778), stable disease (42.2% vs. 54.8%, P = 0.104), and progressive disease (7.6% vs. 15.5%, P = 0.093) were comparable between groups.

**Table 2:** Treatment response based on modified response evaluation criteria in solid tumors criteria.

Variables	Combined group	Rivoceranib-camrelizumab alone group	P value
Complete response	9 (10.8%)	8 (9.5%)	0.778
Partial response	33 (39.8%)	17 (20.2%)	0.006
Stable disease	35 (42.2%)	46 (54.8%)	0.104
Progression disease	6 (7.2%)	13 (15.5%)	0.093
Objective response (complete + partial response)	42 (50.6%)	25 (29.7%)	0.006

### Survival

**Figure 2:**
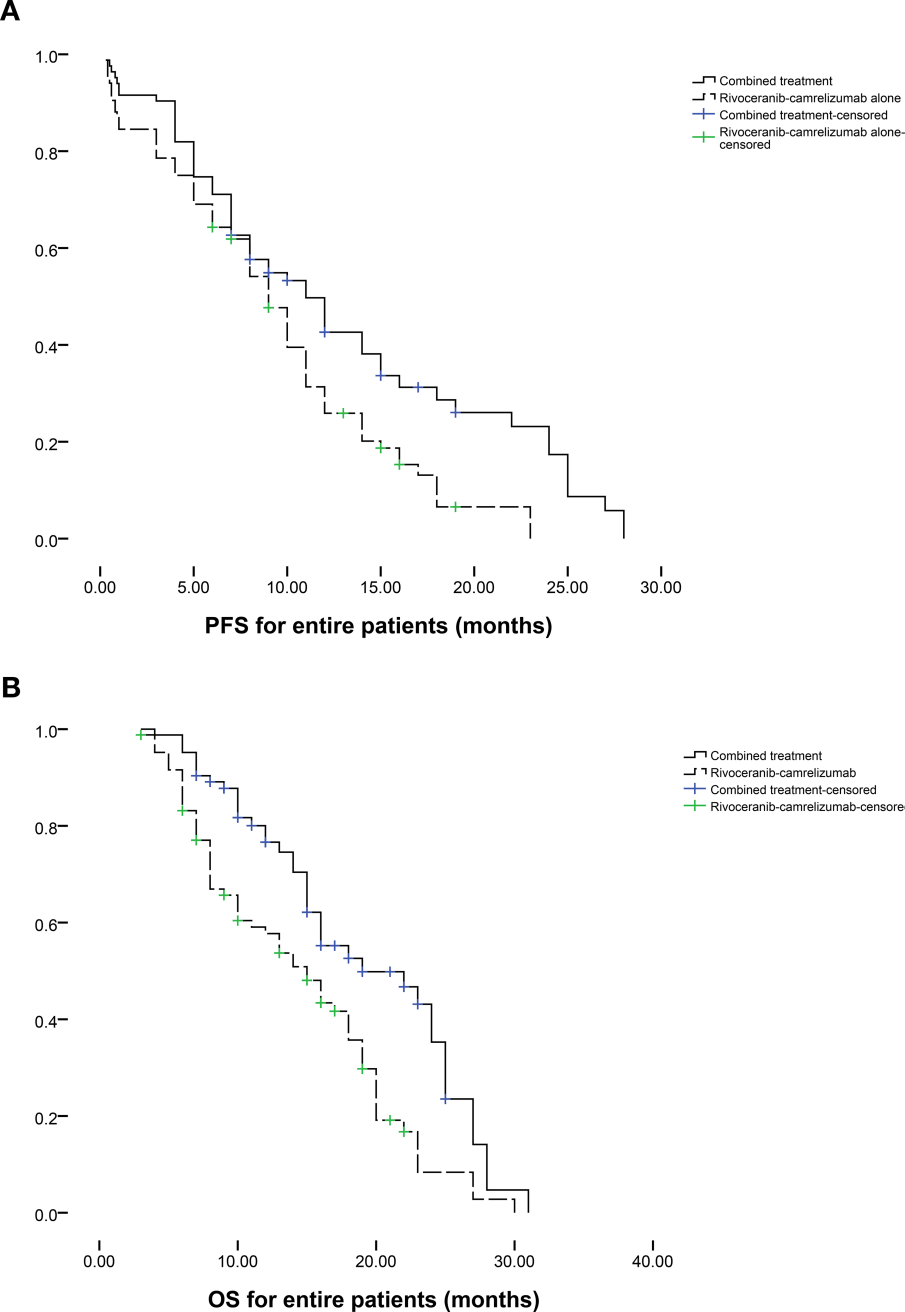
The comparison of **(a)** PFS and **(b)** OS between 2 groups based on the entire patients.

Patients follow-up intervals ranged from 3-31 months (median 12 months). During follow-up, tumor progression occurred in 42 individuals in the combination group and 53 in the rivoceranib-camrelizumab group. The median PFS was markedly longer in the combination group (11.0 vs. 9.0 months, P = 0.008; **Figure 2a**). The one- and two-year rates of PFS were 42.6% and 25.9% for the combination group versus 17.4% and 0% for the monotherapy group, respectively. Among those who progressed in the combination group, 14 underwent microwave ablation, 13 received radioactive seed implantation, and the remainder received conservative care. For patients progressing on rivoceranib-camrelizumab alone, 13 underwent TACE, 12 received hepatic artery infusion chemotherapy, 8 underwent microwave ablation, 6 received radioactive seed placement, and the remaining patients were managed conservatively. Female sex (P = 0.058), ECOG performance status (PS) of 0 (P = 0.089), lower Child-Pugh score (P = 0.033), no extrahepatic metastasis (P = 0.086), and combined therapy (P = 0.011) were correlated with longer PFS in univariate analyses. Multivariate analysis confirmed that combined treatment independently predicted improved PFS (P = 0.012, **Table 3**).

**Table 3:** Cox regression analyses for the progression-free survival.

Variables	Univariate analysis			Multivariate analysis		
	Hazard ratio	95% CI	P value	Hazard ratio	95% CI	P value
Age (y)	0.996	0.976-1.017	0.711			
Gender						
Male	1			1		
Female	0.638	0.401-1.016	0.058	0.663	0.414-1.062	0.087
Hepatitis						
None	1					
B	1.184	0.812-1.725	0.380			
C	0.992	0.353-2.787	0.988			
Hepatocirrhosis	1.271	0.895-1.804	0.181			
ECOG PS						
0	1			1		
1	1.351	0.955-1.912	0.089	1.172	0.813-1.688	0.395
Child-Pugh score	1.181	1.013-1.377	0.033	1.116	0.857-1.301	0.163
ALBI grade						
1	1					
2	0.986	0.686-1.416	0.939			
3	0.761	0.369-1.567	0.458			
AFP value	0.973	0.900-1.052	0.494			
Extra-hepatic metastasis	1.429	0.951-2.150	0.086	1.324	0.867-2.024	0.194
Vascular invasion	0.661	0.403-1.083	0.101			
Tumor number						
1	1					
2	1.080	0.724-1.612	0.706			
≥ 3	1.394	0.868-2.238	0.169			
						
Largest tumor size	0.987	0.940-1.038	0.616			
Treatment groups						
Combined group	1			1		
Rivoceranib-camrelizumab alone group	1.591	1.111-2.280	0.011	1.588	1.107-2.278	0.012

AFP, alpha-fetoprotein; ECOG PS, Eastern Cooperative Oncology Group performance status; HCC, hepatocellular carcinoma.

At the end of follow-up, 41 deaths had occurred in the combination group and 63 in the rivoceranib-camrelizumab group. The median OS was prolonged substantially in the combination group (19.0 vs. 15.0 months, P = 0.001; **Figure 2b**). One- and two-year OS rates were 76.6% and 57.7% for the combined-treatment group versus 35.3% and 8.4% for the single-treatment group. Univariate Cox regression revealed associations between longer OS and ECOG PS 0 (P = 0.051), lower Child-Pugh score (P = 0.021), absence of extrahepatic metastasis (P = 0.045), and combined therapy (P = 0.002). The tumor number ≥ 3 was the predictor of shorter OS (P = 0.029). Multivariate analysis again identified that ECOG PS 0 (P = 0.031), lower Child-Pugh score (P = 0.039), and combined therapy were predictors of prolonged OS (P < 0.001). The tumor number ≥ 3 was the predictor of shorter OS (P = 0.005, **Table 4**).

**Table 4:** Cox regression analyses for the overall survival.

Variables	Univariate analysis			Multivariate analysis		
	Hazard ratio	95% CI	P value	Hazard ratio	95% CI	P value
Age (y)	1.000	0.978-1.023	0.969			
Gender						
Male	1					
Female	0.833	0.513-1.352	0.460			
Hepatitis						
None	1					
B	1.263	0.828-1.926	0.279			
C	0.727	0.173-3.051	0.663			
Hepatocirrhosis	1.132	0.764-1.675	0.537			
ECOG PS						
0	1			1		
1	1.478	0.999-2.186	0.051	1.596	1.043-2.443	0.031
Child-Pugh score	1.241	1.034-1.491	0.021	1.220	1.010-1.473	0.039
ALBI grade						
1	1					
2	1.257	0.827-1.912	0.284			
3	0.858	0.378-1.946	0.713			
AFP value	0.966	0.886-1.053	0.431			
Extra-hepatic metastasis	1.607	1.010-2.556	0.045	1.299	0.795-2.121	0.296
Vascular invasion	0.680	0.383-1.206	0.187			
Tumor number						
1	1			1		
2	1.094	0.685-1.748	0.707	1.147	0.705-1.866	0.580
≥ 3	1.805	1.063-3.066	0.029	2.230	1.276-3.899	0.005
Largest tumor size	1.004	0.951-1.060	0.891			
Treatment groups						
Combined group	1			1		
Rivoceranib-camrelizumab alone group	1.916	1.282-2.863	0.002	2.143	1.419-3.237	< 0.001

AFP, alpha-fetoprotein; ECOG PS, Eastern Cooperative Oncology Group performance status; HCC, hepatocellular carcinoma.

**Figure 3:**
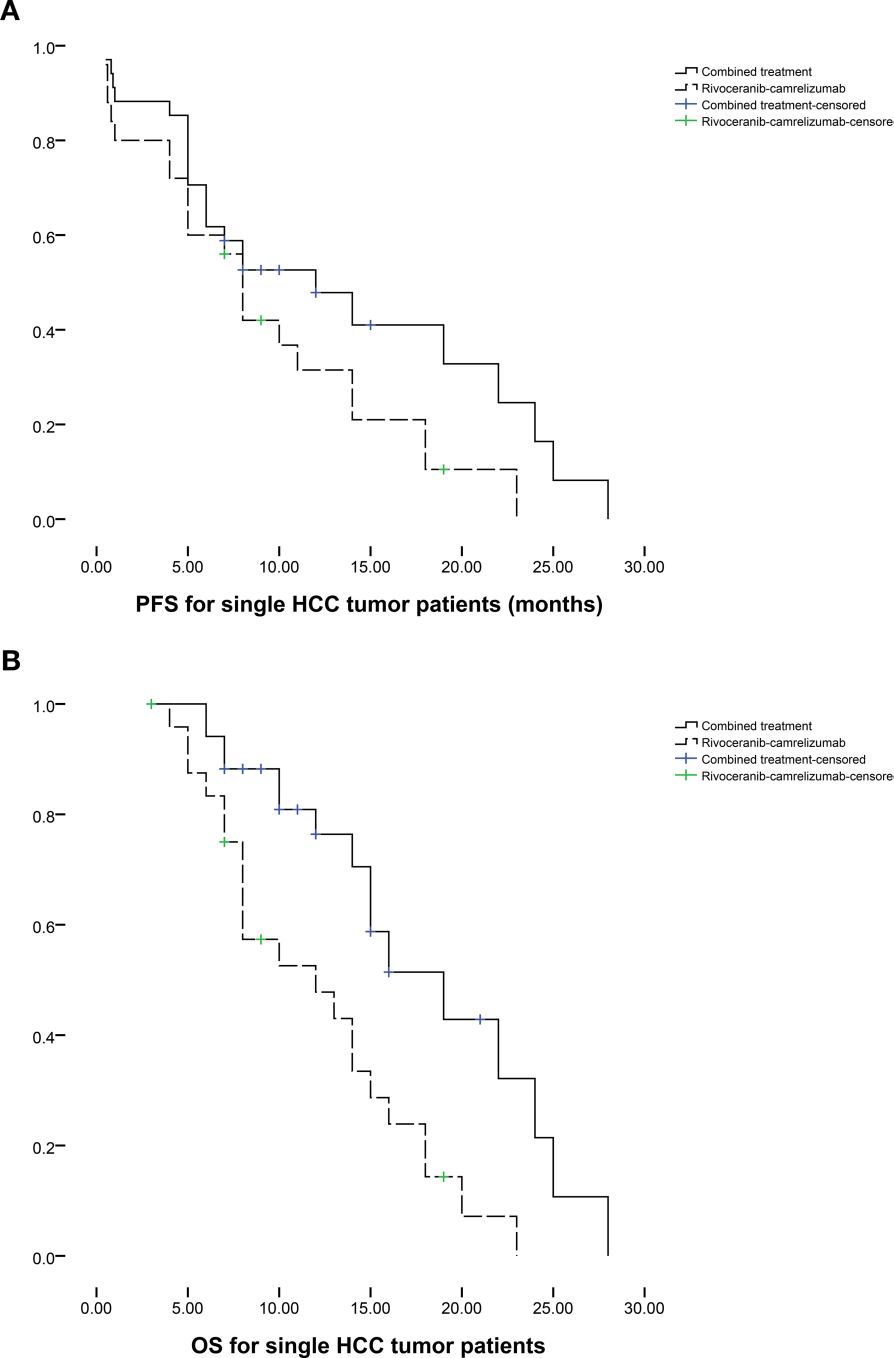
The comparison of **(a)** PFS and **(b)** OS between 2 groups based on the patients with single HCC tumor.

**Figure 4:**
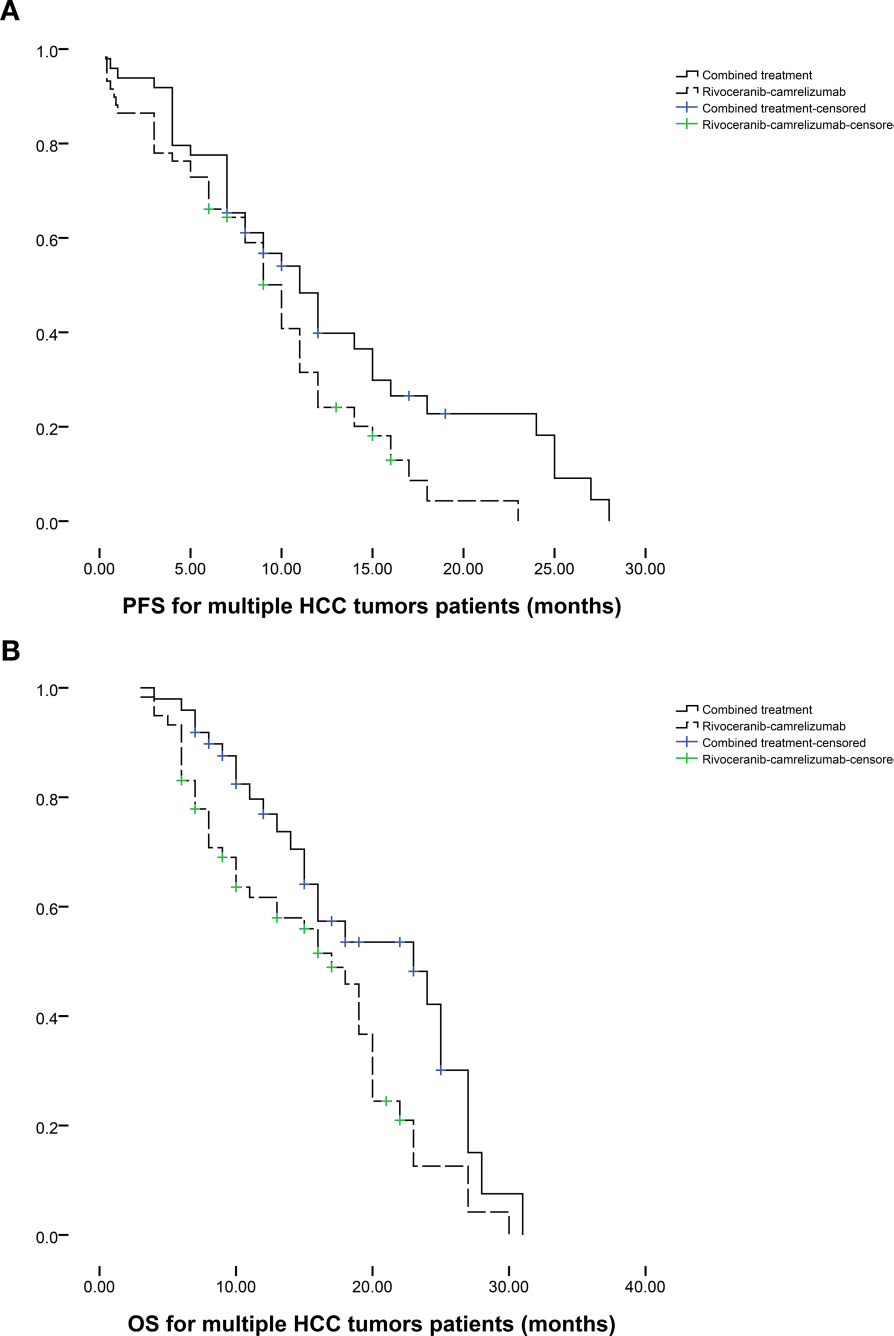
The comparison of **(a)** PFS and **(b)** OS between 2 groups based on the patients with multiple HCC tumors.

Subgroup analyses stratified by tumor number further supported these findings. Among patients with a single HCC lesion (n = 34 in the combination group; n = 25 in the monotherapy group), median PFS favored the combination group (12 vs. 8 months, P = 0.095; **Figure 3a**), while median OS was significantly improved (19 vs. 12 months, P = 0.003; **Figure 3b**). In patients with multiple tumors (n = 49 vs. n = 59), both PFS (11 vs. 10 months, P = 0.039; **Figure 4a**) and OS (23 vs. 17 months, P = 0.019; **Figure 4b**) were prolonged substantially in the combination group.

### Adverse events

Adverse event profiles are summarized in **Table 5**. As expected, TACE-specific post-embolization symptoms occurred only in the combination group but were limited to grade 1-2 severity, and no grade 3-4 post-embolization reactions were observed. The incidence of rivoceranib-camrelizumab-related toxicities was similar between the two cohorts (**Table 5**).

**Table 5:** Adverse events.

Variables	Any grade			Grade 3-4		
	Combined group	Rivoceranib-camrelizumab group	P value	Combined group	Rivoceranib-camrelizumab group	P value
Post-embolization symptoms						
Abdominal pain	42 (50.6%)	Not applicable	Not applicable	0 (0%)	Not applicable	Not applicable
Fever	38 (45.8%)	Not applicable	Not applicable	0 (0%)	Not applicable	Not applicable
Nausea	26 (31.3%)	Not applicable	Not applicable	0 (0%)	Not applicable	Not applicable
Vomiting	10 (12.0%)	Not applicable	Not applicable	0 (0%)	Not applicable	Not applicable
AST increase	11 (13.3%)	Not applicable	Not applicable	0 (0%)	Not applicable	Not applicable
ALT increase	10 (12.0%)	Not applicable	Not applicable	0 (0%)	Not applicable	Not applicable
Rivoceranib-camrelizumab related adverse events						
Hypertension	50 (60.2%)	53 (63.1%)	0.704	20 (24.1%)	22 (26.2%)	0.755
Hand-foot skin reaction	25 (30.1%)	24 (28.6%)	0.826	3 (3.6%)	3 (3.6%)	1.000
Diarrhea	36 (43.4%)	38 (45.2%)	0.808	4 (4.8%)	3 (3.6%)	0.720
Fatigue	27 (32.5%)	25 (29.8%)	0.699	3 (3.6%)	3 (2.4%)	0.682
Dysphonia	21 (25.3%)	22 (26.2%)	0.895	1 (1.2%)	1 (1.2%)	1.000
Decreased appetite	36 (43.4%)	34 (40.5%)	0.704	4 (4.8%)	5 (6.0%)	1.000
Proteinuria	31 (37.3%)	27 (32.1%)	0.480	4 (4.8%)	4 (4.8%)	1.000
Weight decrease	31 (37.3%)	32 (38.1%)	0.921	6 (7.2%)	5 (6.0%)	0.740
Erythra	10 (12.0%)	11 (13.1%)	0.838	1 (1.2%)	1 (1.2%)	1.000
AST increase	4 (4.8%)	5 (6.0%)	1.000	0 (0%)	0 (0%)	Not applicable
ALT increase	4 (4.8%)	6 (7.1%)	0.360	0 (0%)	0 (0%)	Not applicable

## Discussion

Patients diagnosed with BCLC stage C HCC generally have little opportunity for curative resection, making systemic therapy the cornerstone of first-line treatment (3, 9, 10). Historically, the TKIs sorafenib and Lenvatinib represented the standard of care but provided only modest survival advantages (11–13). More recently, PD-1 or PD-L1 checkpoint inhibitors have exhibited favorable efficacy and tolerability profiles in advanced HCC (14, 15).

Rivoceranib is a highly selective, small-molecule TKI directed against VEGFR2. It exerts antitumor activity not only by blocking tumor cell proliferation and neovascularization but also by counteracting the immunosuppressive tumor microenvironment (6). Compared with sorafenib, rivoceranib demonstrates stronger VEGFR2 specificity (16). The humanized IgG4 monoclonal antibody camrelizumab binds with high affinity to PD-1 binding (17). Together, rivoceranib and camrelizumab act synergistically, and their combination has emerged as a novel first-line regimen for advanced HCC (6).

TACE is the most frequently employed locoregional therapy in the context of HCC management (7). However, TACE induces intratumoral hypoxia, which upregulates vascular endothelial growth factor expression and promotes angiogenesis, potentially facilitating tumor progression or metastasis (16, 18). As a result, TACE is often paired with TKIs to offset these effects and enhance antitumor efficacy (9, 16). In the present study, combining TACE with rivoceranib-camrelizumab yielded a substantially higher objective response rate than rivoceranib-camrelizumab alone (50.6% vs. 29.7%, P = 0.006). This finding aligns with Wu et al. (4), who reported superior response rates with TACE plus systemic therapy compared to TACE alone (57.9% vs. 32.6%, P = 0.012) in BCLC stage C patients. However, the result of objective response rate in this present study may be influenced by several factors, such as the retrospective design, potential selection bias, and limited baseline tumor burden characterization. Therefore, further well designed prospective clinical trials should be conducted to validate this result.

Multivariate Cox regression conducted herein confirmed that combined therapy independently predicted longer PFS and OS, supporting a synergistic interaction between TACE and rivoceranib-camrelizumab. Locoregional therapy may potentiate immune checkpoint inhibition by releasing tumor antigens and reshaping the microenvironment, thereby improving immunotherapy responsiveness (19). Here, the median PFS and OS values in the combination group were 11 and 19 months, respectively, which were shorter than the 13.5-month PFS and 24.1-month OS reported in the CHANCE2211 trial (19). This difference likely reflects our exclusive enrollment of BCLC stage C patients, whereas CHANCE2211 included both stage B and C populations.

Furthermore, ECOG PS 0 and lower Child-Pugh score were also the predictors of longer OS, while tumor number ≥ 3 was the predictor of shorter OS in this study. ECOG PS and Child-Pugh score indicate the patients’ body condition and liver function, which are significantly associated with OS. Tumor number is an important factor of tumor burden. Larger tumor burden usually indicates shorter OS (20).

Subgroup analyses further demonstrated consistent benefits of the combined regimen in patients with either single or multiple tumors. Both single and multiple HCC tumor patients exhibited longer PFS and OS in combined group than rivoceranib-camrelizumab alone group. Although PFS improvement did not reach statistical significance in the single-tumor subgroup, the P value of 0.095 also had the tendency of significance. This result may be attributed to the limited sample size rather than absence of effect. Notably, Cox regression did not identify tumor number as a determinant of PFS or OS. These findings indicated that the treatment efficacy of combined treatment may not be influenced by the number of tumors.

Although TACE inevitably introduces post-embolization symptoms, these were mild and transient, with no grade 3-4 events observed (21). The incidence of systemic therapy-related adverse events did not differ significantly between groups, suggesting that adding TACE does not compromise the safety profile of rivoceranib-camrelizumab.

This study has limitations. First, its retrospective design raises potential for selection bias and unmeasured confounders. Prospective randomized trials are warranted to validate our findings. Second, being a single-center investigation limits generalizability, underscoring the need for multicenter studies. Third, the modest sample size may have reduced the power to detect some subgroup differences.

## Conclusion

In summary, our analysis suggests that incorporating TACE into rivoceranib-camrelizumab therapy can enhance clinical efficacy for BCLC stage C HCC without increasing toxicity.

## Data Availability

The raw data supporting the conclusions of this article will be made available by the authors, without undue reservation.
