# Supplementary material for: Transarterial chemoembolization with rivoceranib and camrelizumab for BCLC stage C hepatocellular carcinoma
Source: Front Oncol. 2025 Dec 10;15:1710686. doi: 10.3389/fonc.2025.1710686 (PMC12727550; doi:10.3389/fonc.2025.1710686)
Supplement: Supplementary file 1 [file DataSheet1.doc]

TACE procedures

All TACE procedures were conducted with fluoroscopic guidance and local anesthesia. A 5F catheter (Terumo, Tokyo, Japan) was introduced into the celiac artery, and tumor location was confirmed via angiography. A 2.7F microcatheter (Terumo) was then introduced into the blood-supplying artery for each tumor, and a mix of 5-fluorouracil (150 mg), mitomycin (10 mg), epirubicin (50 mg), and lipiodol (10–20 mL) was introduced for TACE. The microcatheter was then removed, and angiography was repeated with the 5F catheter to confirm TACE efficacy.

If CT/MRI enhancement was identified in treated tumors or new intrahepatic tumor was detected on the contrast-enhanced CT/MRI during follow-up, repeat TACE was performed. When HCC continued to progress after 3 times of TACE or when liver function deteriorated to Child-Pugh C or ECOG PS ≥ 2, TACE was discontinued.

Rivoceranib-camrelizumab treatment

Patients were orally administered rivoceranib 250 mg once daily. Moreover, every two weeks, 200 mg of camrelizumab was given intravenously. Treatment cycles lasted 28 days. According to the dosing guidelines, when adverse events of grade ≥ 3 occurred, patients received a reduced dosage of the drug or discontinued therapy until symptoms resolved or were downgraded to grade 1 or 2.
